# Efficacy and Safety of Manual Small-Incision Cataract Surgery With Trabeculectomy Versus Phacotrabeculectomy in Patients With Glaucoma and Cataract: A Systematic Review and Meta-Analysis

**DOI:** 10.7759/cureus.51025

**Published:** 2023-12-24

**Authors:** El A Andini, Astri Avianty, Herman Herman, Abdul Choliq

**Affiliations:** 1 Ophthalmology, Gunung Jati Regional General Hospital, Cirebon, IDN

**Keywords:** cataract, glaucoma, manual small-incision cataract surgery, phacotrabeculectomy, trabeculectomy

## Abstract

The coexistence of two globally leading causes of blindness, glaucoma and cataracts, is common. Combining trabeculectomy with cataract surgery is a common practice while determining the preferred surgical management for patients is a complex consideration. Therefore, a systematic review and meta-analysis were conducted to compare the efficacy and safety of two surgical procedures, manual small-incision cataract surgery (MSICS) combined with trabeculectomy and *phacotrabeculectomy*. A comprehensive search was performed in PubMed, Science Direct, and Cochrane Library published up to March 2023. Articles not indexed in those databases were also searched. Pooled odds ratios (OR) and mean differences (MD) with corresponding 95% confidence interval (CI) were also retrieved to compare the outcomes estimating efficacy and safety. Biases in selected studies were assessed. A total of seven studies consisting of 352 eyes for MSICS with trabeculectomy and 348 eyes for *phacotrabeculectomy* were included. Postoperative intraocular pressure (IOP) was comparable between the two techniques (MD: -0.45; 95% CI: -1.07 to 0.16; p = 0.15). Postoperative best corrected visual acuity (BCVA) <6/12 (OR: 1.26; 95% CI: 0.62 to 2.53; p = 0.52), complete success (OR: 0.92; 95% CI: 0.51 to 1.67; p = 0.78), and postoperative complications (OR: 1.27; 95% CI: 0.75 to 2.15, p = 0.38) did not differ significantly. This meta-analysis indicated comparable efficacy and safety profile between MSICS with trabeculectomy and *phacotrabeculectomy*. Further high-quality studies are required to confirm these findings.

## Introduction and background

Cataracts and glaucoma are major causes of blindness worldwide, which account for 5.2 million cases and 3.6 million cases, respectively [1]. Future projections estimate that the number will be increased in the coming years [2,3]. The coexistence of cataracts and glaucoma is common among older people, with increasing occurrence after age 60. Cataract is characterized by cloudiness of the lens inside the eye, which can only be treated through surgery. On the other hand, glaucoma is an optic neuropathy that causes irreversible vision loss and is categorized as open-angle or closed-angle based on the configuration of the anterior chamber angle. Surgery is one of the management options, especially for those with advanced glaucoma (uncontrolled intraocular pressure or having maximal medical therapy) [4].

Combining trabeculectomy with cataract surgery is a common practice to manage cataracts associated with glaucoma. This approach provided a more significant decrease in intraocular pressure (IOP), long-term IOP control, fewer postoperative anti-glaucoma medications, less severe damage to the optic nerve, and better postoperative visual acuity [4-6]. However, the procedure can take longer to perform, and there is a risk of complications associated with both cataract surgery and trabeculectomy [5]. The technique of phacoemulsification, as well as manual small-incision cataract surgery (MSICS), have several advantages over other cataract surgery, such as extracapsular cataract extraction, in terms of incision size, rapid recovery, better visual outcome, and lesser intraoperative and postoperative complications [7,8]. MSICS and phacoemulsification showed comparable intra-operative and postoperative complications, but the phacoemulsification group exhibited significantly lower astigmatism [9]. This study aims to evaluate the efficacy and safety of MSICS with trabeculectomy and compare it with phacotrabeculectomy for patients with both cataracts and glaucoma.

## Review

Methods

Search Strategy

This meta-analysis compared clinical outcomes of trabeculectomy combined with MSICS and phacotrabeculectomy in patients with glaucoma and cataract. We combined the terms "phacoemulsification", "phacotrabeculectomy", "manual small-incision cataract surgery", "MSICS", "SICS", "trabeculectomy", "cataract", and "glaucoma" to search the relevant articles published up to March 2023 from several databases such as PubMed, ScienceDirect, and Cochrane Library. Manuscripts not indexed in previously mentioned databases were also searched through website searching and citation tracking.

Study Selection and Eligibility Criteria

We searched for studies matched with the following inclusion criteria: 1) retrospective study, prospective study, or randomized controlled clinical trials in humans comparing MSICS-trabeculectomy and phacoemulsification-trabeculectomy are included; 2) participants with any type of glaucoma and coexisting cataract; 3) outcomes had to be clearly reported. The primary outcome was postoperative IOP. In addition, we also evaluated visual acuity, surgical success for trabeculectomy, and the incidence of postoperative complications.

The visual acuity cut-off of 6/12 was chosen since it is classified as visual impairment, according to the World Health Organization [10]. Complete success was defined as an IOP level below 21 mmHg without requiring additional anti-glaucoma medications (AGM) or surgery. We examined the proportion of eyes having postoperative adverse events such as moderate to severe uveitis with fibrinous exudate, fibrinous reaction, transient or persistent hypotony, descemet detachment, hyphema, secondary glaucoma, cystoid macular edema, bleb infiltrate or blebitis, choroidal detachment, and endophthalmitis.

Two reviewers independently screened the studies, employing pre-established criteria. We independently confirmed study eligibility according to the criteria and judged the risk of bias in each article. Collected data included demographic characteristics and baseline and postoperative measures.

Quality Assessment

The Newcastle-Ottawa Quality Assessment Scale (NOS) criteria list was used to assess the risk of bias in the trials [11]. The criteria were scored as one star for each number of Selection (0-4) and Exposure (0-3) categories. Maximum two stars for Comparability (0-2). A study meeting at least 7 of the 12 items was considered a high-quality study. The maximum score from NOS is 9. A study with a score in the range of 4-6 is considered a high risk of bias, and a score of 0-3 is a very high risk of bias study.

Statistical Analysis

All eligible studies were reviewed quantitatively. STATA 17.0 (StataCorp, Texas, USA) was used for all statistical analyses. Pooled mean difference (MD) was used to report continuous outcomes, and odds ratio (OR) was used for dichotomous outcomes, with 95% confidence intervals (CI) reported for all outcomes. A p-value of less than 0.05 was considered statistically significant. The I2 index was used to determine heterogeneity among the results, with significant heterogeneity set at the I2 > 50% level. The funnel plot was used to detect any other source of publication bias. However, we did not have enough studies (n ≥ 10) to analyze the funnel plot of the intervention effect estimates for evidence of asymmetry.

Result

**Figure 1:**
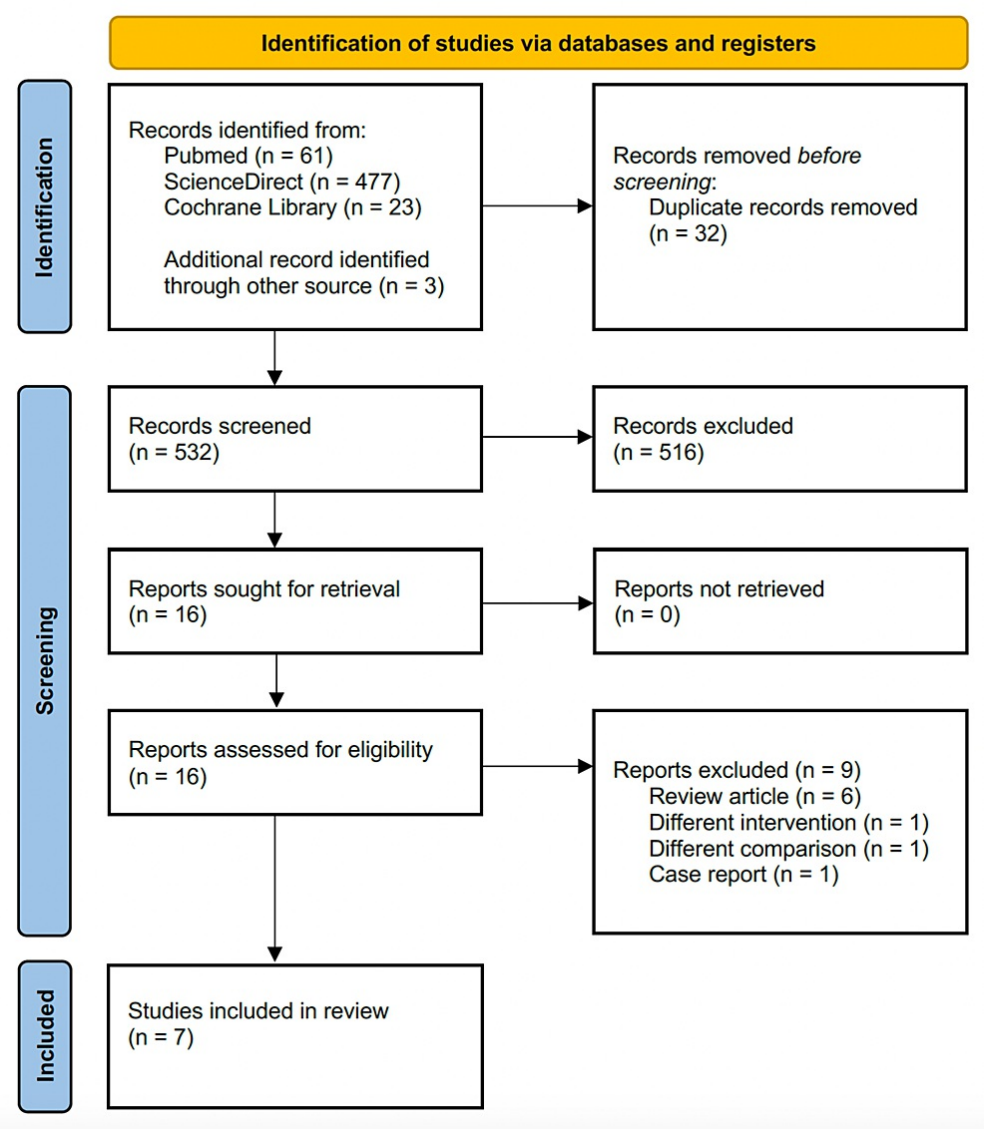
PRISMA flow diagram of the study selection process PRISMA: Preferred Reporting Items for Systematic Reviews and Meta-Analyses

We found a total of 561 relevant studies through our search strategy. We also considered three hand-picked articles. After eliminating duplicate studies, we reviewed the remaining 532 based on their title and abstracts. Sixteen articles met our criteria and were assessed for eligibility through a full-text review. Eventually, we identified seven appropriate studies for our qualitative and quantitative review. The selection process is presented in Figure 1.

Study Characteristics

We reviewed seven retrospective studies comparing (a) Manual small-incision cataract surgery with trabeculectomy (n = 352 eyes) and (b) Phacoemulsification with trabeculectomy (n = 348) eyes [12-18]. No prospective studies were found from the study selection process. The summary of the study baseline characteristics and quality assessment is presented in Table 1. All the studies included in the analysis reported both follow-up and baseline data, but none provided information on whether they adjusted for confounders in their analysis. One study exhibited a statistically significant difference in mean age, rendering it unable to meet one of the selection criteria in NOS. As a result, this study was deemed to have a high risk of bias.

**Table 1:** Characteristics and risk of bias assessment of involved studies M: MSICS-trabeculectomy; P: Phacotrabeculectomy; NOS: Newcastle-Ottawa Scale; BCVA: Best-corrected visual acuity; IOP: intraocular pressure; POAG: primary open-angle glaucoma; PACG: primary angle closure glaucoma; CACG: chronic angle closure glaucoma; PXF: pseudoexfoliation

Studies	Location	Study type	Sample size (M vs. P)	Mean age (years) (M vs. P)	Mean follow-up (months) (M vs. P)	Glaucoma type	Preoperative IOP (mmHg) (M vs. P)	Outcome measures	NOS
Thomas et al., 2003 [12]	India	Retrospective study	86 vs. 78	55.1 ± 7.5 vs. 54.7 ± 7.2	11 ± 4.8 vs. 17 ± 7.8	POAG, PACG	31.8 ± 9.4 vs. 32.5 ± 8.4	IOP, complication, visual acuity, success rate, bleb morphology	7
Gandasubrata et al., 2005 [13]	Indonesia	Retrospective study	11 vs. 10	67.6 ± 7.7 vs. 57.7 ±12	3	POAG, CACG	38.8 ± 12.65 vs. 32.3 ± 6.25	IOP, visual acuity	6
Mittal et al., 2008 [14]	India	Retrospective study	55 vs. 52	64.13 (41-80) vs. 66.11 (49-83)	36.1 ±16.8 vs. 42 ±16.8	Open-angle, angle closure, combined, PXF	19.89 ± 7.47 vs. 18.0 ± 6.45	IOP, visual acuity, success rate, complication, antiglaucoma medication, cup-disk ratio	7
Khandelwal et al., 2015 [15]	India	Retrospective study	53 vs. 22	61.1 ± 8.76 vs. 60.36 ± 7.05	13.73	POAG, PACG	26.5 ±5 vs. 25.2 ±5	IOP, complication, success, visual acuity	7
Lone et al., 2019 [16]	India	Retrospective study	25 vs. 25	69.1 ± 4.8 vs. 69.9 ± 4.76	6	PXF	26.4 ± 3.38 vs. 26.4 ± 3.66	IOP, BCVA, complication	7
Ramyashri et al., 2020 [17]	India	Retrospective study	82 vs. 64	71 ± 10.3 vs. 68 ± 7.8	1 ± 0.9 vs. 0.75 ± 0.57	PXF	26 ± 10.3 vs. 23 ± 13.9	IOP, success rate, complication, visual acuity, surgery time	7
Mansoori et al., 2020 [18]	India	Retrospective study	40 vs. 97	64.47 ± 13.68 vs. 66.18 ± 8.16	18.6 ± 7.7	POAG, PACG	22.28 ± 8.94 vs. 23.79 ± 9.7	IOP, visual acuity, antiglaucoma medication, success rate	7

Intraocular Pressure

**Figure 2:**
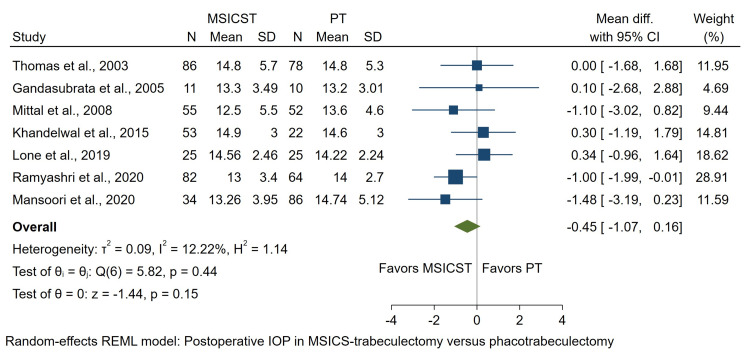
Forest plot of the postoperative IOP at 1-2 months follow-up in eyes undergoing MSICS-trabeculectomy versus phacotrabeculectomy MSICST: manual small-incision cataract surgery-trabeculectomy, PT: phacoemulsification-trabeculectomy, IOP: intraocular pressure [12-18]

A total of seven studies were examined to compare postoperative IOP. The studies had varying follow-up periods, ranging from one day to three years. We used reported postoperative IOP of 1-2 months in all studies to be analyzed. The test for heterogeneity was not significant (I2 = 12.22%, p = 0.44). The analysis showed no significant difference between the two groups regarding postoperative IOP (MD: -0.45; 95% CI: -1.07 to 0.16; p = 0.15), as shown in Figure 2.

In the study by Ramyashri et al., the final IOP at the last visit was 12±3.2 for the MSICS-trabeculectomy group and 14±1.8 for the phacotrabeculectomy group [17]. Lone et al. reported a 6-month IOP of 15.64 ± 2.43 for MSICS-trabeculectomy compared to 17.19 ± 2.56 for phacotrabeculectomy [16]. Khandelwal et al. observed IOP values of 14.9 ± 3 versus 14.6 ± 3 in the 12-18 months range during the final follow-up [15]. Thomas et al. documented 12-month IOP values of 16.0 ± 5.9 mmHg for MSICS-trabeculectomy and 15.0 ± 5.7 for phacotrabeculectomy [12]. In the study by Mittal et al., the IOP was 13.73 ± 3.8 (mean follow-up of 36.1 months) and 13.87 ± 3.5 (mean follow-up of 42.0 months) [14]. Finally, Mansoori et al. reported the last follow-up visit IOP as 14.08 ± 4.12 for MSICS-trabeculectomy and 13.9 ± 2.98 for phacotrabeculectomy within a range of 12-40 months [18].

Two studies reported the mean reduction of IOP. In the first study, the mean IOP reduction was 17.1 ± 10 mmHg in eyes with MSICS-trabeculectomy and 17.7 ± 9.3 mmHg in the phacotrabeculectomy group [12]. The other study reported 14.1 ± 4.12 mmHg IOP reduction in the MSICS-trabeculectomy group and 13.9 ± 2.98 mmHg in phacotrabeculectomy, which did not differ significantly [18].

Visual Acuity

**Figure 3:**
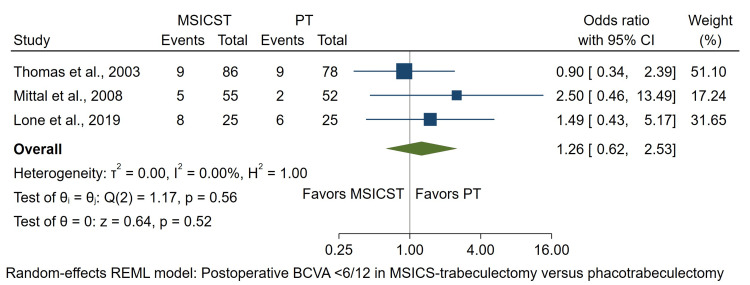
Forest plot of the postoperative BCVA <6/12 in eyes undergoing MSICS-trabeculectomy versus phacotrabeculectomy MSICST: manual small-incision cataract surgery-trabeculectomy, PT: phacoemulsification-trabeculectomy, BCVA: best corrected visual acuity [12,14,16]

In three studies, postoperative visual acuity was compared categorically between the two groups [12,14,16]. The analysis was conducted using data from these studies, in which the events were postoperative BCVA <6/12 at a follow-up range of 2 months to 1 year. The test for heterogeneity was not significant (I2 = 0%, p = 0.56). The meta-analysis showed that the difference between the groups was not statistically significant (OR: 1.26; 95% CI: 0.62 to 2.53; p = 0.52), as depicted in Figure 3.

The other four studies were not included in the visual acuity analysis because of different outcome categorizations and definitions. One showed that most patients had preoperative visual acuity <0.1, which improved to 0.1-0.3 postoperatively in both groups in 3 months [13]. In Ramyashri et al., 96% of eyes in the MSCIS-trabeculectomy group and 95% of eyes in the phacotrabeculectomy group increased >3 line Snellen charts without any of the eyes experiencing a decline in BCVA [17]. The other study reported a >2 Snellen line improvement of 49.1% in MSICS-trabeculectomy and 54.5% in phacotrabeculectomy in 12 months [15]. Mansoori et al. showed mean postoperative LogMAR BCVA 0.21 ± 0.33 in MSICS-trabeculectomy and 0.22 ± 0.31 in phacotrabeculectomy [18]. Those studies also reported that the postoperative BCVA or visual acuity improvement did not differ significantly between the two types of combined surgeries.

Complete Success

**Figure 4:**
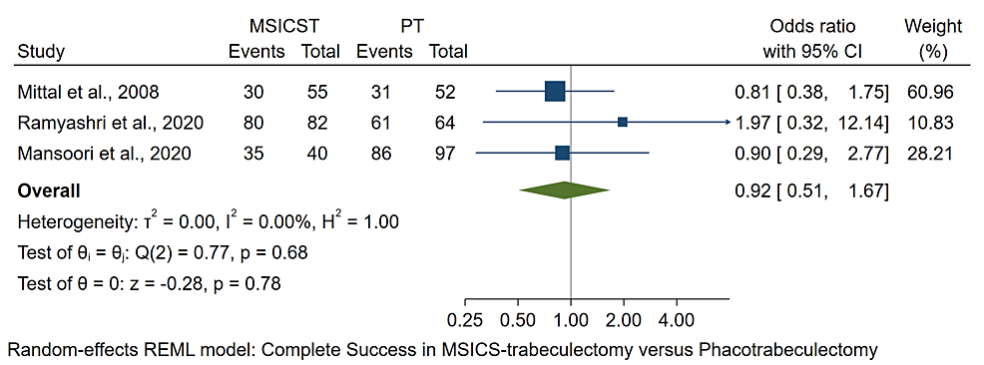
Forest plot of the complete success number in eyes undergoing MSICS-trabeculectomy versus phacotrabeculectomy MSICST: manual small-incision cataract surgery-trabeculectomy, PT: phacoemulsification-trabeculectomy [14,17,18]

The number of complete surgical successes was examined from three studies [14,17,18]. The results showed no significant difference in complete success rate between the two surgeries (OR: 0.92; 95% CI: 0.51 to 1.67; p = 0.78), as demonstrated in Figure 4. The analysis did not include two other studies due to different outcome definitions. Those studies also showed no significant difference in surgical success between the two interventions [12,15].

Postoperative Complications

**Figure 5:**
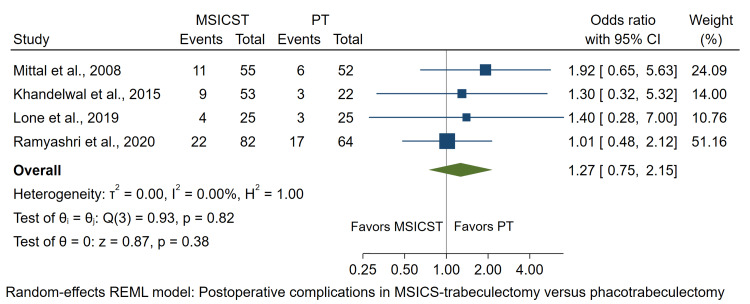
Figure 5. Forest plot of the postoperative complications in eyes undergoing MSICS-trabeculectomy versus phacotrabeculectomy MSICST: manual small-incision cataract surgery-trabeculectomy. PT: phacoemulsification-trabeculectomy [14-17]

Postoperative complications were obtained from four studies. The number included in the analysis was the number of eyes with postoperative complications. The analysis showed no significant difference in the incidence of postoperative complications between the two surgeries (OR: 1.27, 95% CI: 0.75 to 2.15, p = 0.38), as summarized in Figure 5.

Two other studies reported the incidence of each complication. Hyphema was seen in 4.7% of the MSICS-trabeculectomy and 3.9% in the phacotrabeculectomy [12]. Tight blebs were examined in both groups, 5.5% versus 9.6%. Posterior capsule opacification was most reported in the third postoperative year, which affected 49.1% of the MSICS-trabeculectomy and 40.4% of the phacotrabeculectomy group [14].

Anti-Glaucoma Medication and Cup-Disk Ratio

Mansoori et al. reported that the preoperative mean number of anti-glaucoma medications was 1.95 ± 0.96 in the MSICS-trabeculectomy and 1.84 ± 1.0 in the phacotrabeculectomy group. Those numbers were reduced to 0.025 ± 0.7 in MSICS-trabeculectomy and 0.03 ± 0.8 in phacotrabeculectomy postoperatively [18]. In another study, the mean number of anti-glaucoma medications declined in the MSICS-trabeculectomy group but slightly increased in the phacotrabeculectomy group [14]. The mean medication changes between the two groups after surgery were not statistically significant [14,18].

The vertical cup-to-disc ratio increased significantly in both groups during the follow-up period. It increased from 0.773 ± 0.147 to 0.790 ± 0.149 in the eyes with MSICS-trabeculectomy and from 0.722 ± 0.143 to 0.749 ± 0.124 in the phacotrabeculectomy group. However, there was no significant difference in the change in cup-to-disc ratio after surgery between the two groups (p = 0.47) [14].

Discussion

This current systematic review and meta-analysis explored the efficacy and safety of two combined glaucoma and cataract procedures, MSICS-trabeculectomy and phacoemulsification-trabeculectomy, the first study to our knowledge. All included studies reported a significant reduction in the mean intraocular pressure (IOP) after the surgery in both groups. Removing the lens causes a larger opening of the angle, which can be used to remove the pupillary block and stop the progression of angle-closure glaucoma. A new drainage pathway for aqueous humor is constructed, increasing the aqueous outflow. Moreover, it lessens the likelihood of anterior chamber absence or flatness, which typically follows trabeculectomy surgery alone. The previous meta-analysis discovered that combined surgery had a significantly greater IOP lowering effect than cataract surgery alone and was comparable with trabeculectomy alone [6,7,19]. In terms of two different combined surgeries, MSICS-trabeculectomy and phacotrabeculectomy, our findings revealed no significant difference in postoperative IOP between them. 

Previous studies showed combined surgery provides better outcomes in visual acuity than two-stage surgery and fewer AGM needed postoperatively than phacoemulsification only [6,7]. The studies involved in this analysis showed a tremendous visual acuity improvement after both surgery groups. However, the comparison of prevalence of complete success, anti-glaucoma medication, and postoperative BCVA between the two groups did not differ significantly. The result is in accordance with a previous study that showed that uncorrected visual acuity (UCVA) and BCVA of 6/18 cut-off were comparable between two cataract surgeries, MSICS and phacoemulsification. A slight difference for the 6/9 UCVA cut-off was seen favoring phacoemulsification over MSICS [5].

This recent analysis also showed no significant difference in postoperative complications between the two combined procedures. The result can be explained by a previous meta-analysis comparing MSICS and phacoemulsification, which showed no difference in intraoperative and postoperative complications. However, the phacoemulsification group reported statistically significantly less astigmatism [5]. For consideration, combined surgery showed a lower risk of complication than two-stage procedures [7]. Conversely, combined procedures reported a higher risk of complications than phacoemulsification alone [6]. Ramyashri et al. showed no significant difference in surgery time between MSICS-trabeculectomy and phacotrabeculectomy [17]. However, the lower average surgery time, lower surgery cost, and less equipment needed led MSICS to offer some advantages in resource-limited settings and a backlog of blindness conditions other than phacoemulsification. Even in developed countries, MSICS could be a suitable choice for dense cataracts where the posterior capsule cannot be seen, for hard cataracts where phacodonesis is present, and for inexperienced surgeons considering lesser postoperative complications among them [5].

Limitation

The present study had some limitations. First, all of our data is based on retrospective studies (non-randomized studies), resulting in a lower level of evidence for our conclusions. Since the data were obtained retrospectively, the assignment of patients for each intervention was based on consideration of the best option for the patient. Second, differences in definitions introduce uncertainty when summarizing the conclusion. Third, we examined different subtypes of glaucoma and cataracts, which may introduce some uncertainty in the results. Fourth, we used any trabeculectomy procedures with or without anti-metabolite. Different procedure methods could be handled by performing separate subanalyses, which we did not conduct due to a lack of studies. Also, all the studies come from the Asian population, which might not represent the world's population. Therefore, our results should be interpreted wisely.

## Conclusions

Our study showed comparable efficacy and safety profile between MSICS with trabeculectomy and phacotrabeculectomy. There was no significant difference in postoperative intraocular pressure, postoperative BCVA, complete success number, and postoperative complication between the two combined procedures in this study. However, both techniques have advantages and indications concerning personalized settings and conditions. Further high-quality and better-designed studies are needed to explore and confirm the comparison between the two groups.
